# Numerical solution of multi-dimensional time-fractional diffusion problems using an integral approach

**DOI:** 10.1371/journal.pone.0304395

**Published:** 2024-09-23

**Authors:** Muhammad Nadeem, Shamoona Jabeen, Fawziah M. Alotaibi, Yahya Alsayaad

**Affiliations:** 1 School of Mathematics and Statistics, Qujing Normal University, Qujing, China; 2 Department of Mathematics, University of Science and Technology, Bannu, KPK, Pakistan; 3 Department of Mathematics, Turabah University College, Taif University, Taif, Saudi Arabia; 4 Department of Physics, Hodeidah University, Al-Hudaydah, Yemen; Industrial University of Ho Chi Minh City, VIET NAM

## Abstract

This paper presents a significant scheme to drive the numerical solution of multi-dimensional diffusion problems where the fractional derivatives are taken in Caputo sense. The Mohand homotopy integral transform scheme (MHITS) is the composition of Mohand integral transform (MIT) and the homotopy perturbation scheme (HPS) which can be used to investigate the numerical solution in the form of convergence series. This approach does not require any presumptions, limitations on elements, or any other hypothesis. The primary objective of this strategy is to perform its direct implementation to the recurrence relation. This method produces results in the form of a convergent series, which accurately predicts the exact results. Graphical results and plot error distribution show an excellent agreement between MHITS results and the exact solution.

## 1 Introduction

Fractional partial differential equations (FPDEs) have been noticed in several fields of engineering, including astronomy, engineering and other scientific fields. In recent, many physical experiments showed that fractional orders behave better performance with the experimental data than the integer order derivatives [1–3]. The differential systems with fractional orders have suddenly started much attraction in the process of creating models and studying dynamical systems. Many researchers have focused a great devotion and appreciation due to its various phenomena in nature. The classical theory of fractional calculus is widely recognized as an essential component of the primary curricula in multiple disciplines of scientific research, such as kinematics and hydrodynamics mechanics [4], fractal geometry [5], bioengineering [6], electrodynamics [7], and fluid dynamics [8]. Various computational and analytical schemes have been studied for obtaining the solution of FPDEs such as Homotopy perturbation transform scheme [9], Variational iteration method [10], Optimal Homotopy asymptotic method [11], Reduced differential transform method [12], Homotopy analysis technique [13], Matrix of integration method [14], Shifted Legendre polynomials [15], *q*-homotopy analysis transform method [16], Generalized Mittag-Leffler function method [17], and Modified Galerkin algorithm [18].

The study of time-fractional diffusion problem has attained more consideration due to its density dynamics in a material undergoing diffusion and defining the mechanisms in a diffusive sense. Various researchers studied these problems with numerous analytical schemes and found that the solution to nonlinear fractional diffusion problems is still a challenging task. Akbarzade and Langari [19] presented the study of analytical schemes to derive the numerical results of a three-dimensional heat problem. Later, Kumar et al. [20] implement the idea of HPS and produce the series solutions for the multi-dimensional diffusion problems of fractional order. He showed that HPS has strong performance for the nonlinear fractional problems. The strategy of variational iteration was studied by Prakash and Kumar [21] to derive the approximate results for the presented model. Mahalakshmi et al. [22] considered a homotopy analysis strategy to compute the series solutions for thermal absorbtion problem in two-dimensional form. Nourazar and Golsha [23] presented the modification of HPS for the analytical solution of Cauchy reaction-diffusion problem. Agarwal and El-Sayed [24] demonstrated the computational results for the diffusion problems of fractional order under the study of finite difference scheme and Chebyshev collocation approach. A recent study on multi-dimensional diffusion problems can be tracked in [25]. However, these schemes are based on some assumptions and somewhere on the restriction of variables during the formulation of these ideas.

In this article, we construct a strategy with the combination of MIT and HPS for the numerical treatment of the multi-diffusion equation of time-fractional order. This scheme is suitable for approximate results of fractional differential problems. Since MIT is effective in operating the linear terms only whereas the homotopy perturbation scheme computes the results in terms of series very swiftly. The fractional order of the differential problem determines the rate of convergence. The obtained series quickly exposes the findings, and we note that the computational series rapidly approaches exact results with a minimal number of iterations. This method is not dependent on assumptions, restricted variables, or linearization. This work is organized as; we discuss a few concepts of Mohand transform in Section (2). The formulation of MHITS for the proposed model is explained in Section (3). We offer four applications to verify the credibility and dependability of MHITS in Section (4) and finally, we conclude this study in Section (5).

## 2 Fundamental concept of MIT and fractional calculus

This section provides an overview of certain properties of MIT that are crucial to the creation of our propose strategy.

**Definition 2.1** Let *ϑ*(*ξ*) be a function such as [26]

$$\begin{array}{c} L\left\{\vartheta \left(\xi \right)\right\}=R\left(\nu \right)=\int_{0}^{\infty }\vartheta \left(\xi \right)e^{-\nu \xi }d\xi , \end{array}$$

and *v* exists for all in a domain *D*, then *R*(*ν*) is Laplace transform of function *ϑ*(*ξ*).

**Definition 2.2** Let *α* be the fractional order of function *ϑ*(*ξ*), then right-modified Riemann-Liouville derivativ is [27]

$$\begin{array}{c} \frac{\partial^{\alpha }\vartheta }{\partial \xi^{\alpha }}=\frac{1}{\Gamma (1-\alpha )}\frac{d}{d\xi }\int_{0}^{\xi }{\left(\xi -\delta \right)}^{-\alpha }\vartheta \left(\delta \right)d\xi ,\;\;0<\alpha <1. \end{array}$$

**Definition 2.3** The fractional derivative of *ϑ*(*ξ*) in Caputo form is described as [28]

$$\begin{array}{c} D_{\xi }^{\alpha }\vartheta \left(\delta ,\xi \right)=\frac{1}{\Gamma (n-\alpha )}\int_{0}^{\xi }{\left(\xi -\varrho \right)}^{n-\alpha -1}\vartheta^{n}\left(\rho \right)\;\partial \rho ,\;n-1<\alpha <n,\;n\in N,\;\xi >0,\;\rho \geq -1. \end{array}$$

**Definition 2.4** Mohand and Mahgoub [29] construct a concept of MIT which is expressed as

$$\begin{array}{c} \text{M}\left\{\vartheta \left(\xi \right)\right\}=R\left(\omega \right)=\omega^{2}\int_{0}^{\xi }\vartheta \left(\xi \right)e^{-\omega \xi }d\xi ,k_{1}\leq \omega \leq k_{2}. \end{array}$$

Since, *R*(*ω*) is denoted as MIT of *ϑ*(*ξ*), then

$$\begin{array}{c} {\text{M}}^{-1}\left\{R\left(\omega \right)\right\}=\vartheta \left(\xi \right), \end{array}$$

here **M**^−1^ is nominated as the inverse MIT.

**Properties 1** Some properties of the Mohand transform for a differential function *ϑ*(*ξ*) are as follows,

a) **M**{*ϑ*′(*ξ*)} = *ωR*(*ω*) − *ω*^2^*ϑ*(0).
b) **M**{*ϑ*″(*ξ*)} = *ω*^2^*R*(*ω*) − *ω*^3^*ϑ*(0) − *ω*^2^*ϑ*′(0).
c) **M**{*ϑ*^*n*^(*ξ*)} = *ω*^*n*^*R*(*ω*) − *ω*^*n*+1^*ϑ*(0) − *ω*^*n*^*ϑ*′(0) − ⋯ − *ω*^*n*^*ϑ*^*n*−1^(0).

**Definition 2.5** The fractional order of Mohand transform is explained as [30]

$$\begin{array}{c} \text{M}\left\{\vartheta^{\alpha }\left(\xi \right)\right\}=\omega^{\alpha }R\left(\omega \right)-\sum_{k=0}^{n-1}\frac{\vartheta^{k}\left(0\right)}{\omega^{k}-\left(\alpha +1\right)},\;\;\;0<\alpha \leq n. \end{array}$$

## 3 Idea of MHITS

This segment introduces the formulation of MHITS for obtaining the iterative series of multi-dimensional fractional diffusion problems. The construction of this strategy is very straightforward and performs strong agreement among the MHITS results and the precise results. Consider a fractional problem of order *α* such as

$$\begin{array}{c} D_{\xi }^{\alpha }\vartheta \left(\delta ,\xi \right)+L_{1}\vartheta \left(\delta ,\xi \right)+L_{2}\vartheta \left(\delta ,\xi \right)=g\left(\delta ,\xi \right), \end{array}$$ (1)

$$\begin{array}{c} \vartheta (\delta ,0)=h(\delta ), \end{array}$$ (2)

in which *α* represent the fractional order of *ϑ*(*δ*, *ξ*) towards the route of *δ* and *ξ*. Also, *L*_1_ and *L*_2_ represent the linear and the nonlinear differential operators and *g*(*δ*, *ξ*) is a known component.

**Step 1**: Employing MIT on Eq (1), we get

$$\begin{array}{c} \text{M}[D_{\xi }^{\alpha }\vartheta \left(\delta ,\xi \right)+L_{1}\vartheta \left(\delta ,\xi \right)+L_{2}\vartheta \left(\delta ,\xi \right)]=\text{M}[g(\delta ,\xi )], \end{array}$$ (3)

which means

$$\begin{array}{c} \omega^{\alpha }[R(\omega )-\omega \vartheta (0)]=-\text{M}[L_{1}\vartheta \left(\delta ,\xi \right)+L_{2}\vartheta \left(\delta ,\xi \right)]+\text{M}[g(\delta ,\xi )], \end{array}$$

Thus, *R*(*ω*) obtained as

$$\begin{array}{c} R\left(\omega \right)=\omega \vartheta \left(0\right)-\frac{1}{\omega^{\alpha }}\text{M}[L_{1}\vartheta \left(\delta ,\xi \right)+L_{2}\vartheta \left(\delta ,\xi \right)+g\left(\delta ,\xi \right)]. \end{array}$$

Using condition ((2)), it yields

$$\begin{array}{c} R\left(\omega \right)=\omega h\left(\delta \right)-\frac{1}{\omega^{\alpha }}\text{M}[L_{1}\vartheta \left(\delta ,\xi \right)+L_{2}\vartheta \left(\delta ,\xi \right)+g\left(\delta ,\xi \right)]. \end{array}$$

**Step 2**: Utilizing inverse MIT, we determine

$$\begin{array}{c} \vartheta \left(\delta ,\xi \right)=G\left(\delta ,\xi \right)-{\text{M}}^{-1}[\frac{1}{\omega^{\alpha }}\text{M}[L_{1}\vartheta \left(\delta ,\xi \right)+L_{2}\vartheta \left(\delta ,\xi \right)]], \end{array}$$ (4)

in which

$$\begin{array}{c} G\left(\delta ,\xi \right)={\text{M}}^{-1}[\omega h\left(\delta \right)-\frac{1}{\omega^{\alpha }}\text{M}\{g(\delta ,\xi )\}]. \end{array}$$

**Step 3**: Let the general solution of Eq (1) is

$$\begin{array}{c} \vartheta \left(\delta ,\xi \right)=\sum_{n=0}^{\infty }p^{n}\vartheta_{n}\left(\delta ,\xi \right), \end{array}$$ (5)

and

$$\begin{array}{c} L_{2}\vartheta \left(\delta ,\xi \right)=\sum_{n=0}^{\infty }p^{n}H_{n}\vartheta \left(\delta ,\xi \right), \end{array}$$ (6)

where *p* ∈ [0, 1] is small homotopy parameter and *ϑ*_0_(*δ*, *ξ*) is starting point of Eq (1).

**Step 4**: We have the following iteration strategy to obtain the He’s polynomials

$$\begin{array}{c} H_{n}\left(\vartheta_{0}+\vartheta_{1}+\cdots +\vartheta_{n}\right)=\frac{1}{n!}\frac{\partial^{n}}{\partial p^{n}}(L_{2}(\sum_{i=0}^{\infty }p^{i}\vartheta_{i}){)}_{p=0},\;\;\;\;n=0,1,2,\cdots . \end{array}$$

Combining the Eqs (5), (6) and (4) can be written as

$$\begin{array}{c} \sum_{n=0}^{\infty }p^{n}\vartheta_{n}\left(\delta ,\xi \right)=G\left(\delta ,\xi \right)-p{\text{M}}^{-1}[\frac{1}{\omega^{\alpha }}\text{M}\{L_{1}(\sum_{n=0}^{\infty }p^{n}\vartheta_{n}\left(\delta ,\xi \right))+\sum_{n=0}^{\infty }p^{n}H_{n}\vartheta_{n}\left(\delta ,\xi \right)\}]. \end{array}$$

By comparing the corresponding parts of *p*, we obtain

$$\begin{array}{c} \begin{array}{cc} p^{0} & :\vartheta_{0}\left(\delta ,\xi \right)=G\left(\delta ,\xi \right),\\ p^{1} & :\vartheta_{1}\left(\delta ,\xi \right)=-{\text{M}}^{-1}[\frac{1}{\omega^{\alpha }}\text{M}\{L_{1}\vartheta_{0}\left(\delta ,\xi \right)+H_{0}\}],\\ p^{2} & :\vartheta_{2}\left(\delta ,\xi \right)=-{\text{M}}^{-1}[\frac{1}{\omega^{\alpha }}\text{M}\{L_{1}\vartheta_{1}\left(\delta ,\xi \right)+H_{1}\}],\\ p^{3} & :\vartheta_{3}\left(\delta ,\xi \right)=-{\text{M}}^{-1}[\frac{1}{\omega^{\alpha }}\text{M}\{L_{1}\vartheta_{2}\left(\delta ,\xi \right)+H_{2}\}],\\ & \vdots \end{array} \end{array}$$ (7)

**Step 5**: Hence, we can produce the following series in the form of *p* such as

$$\vartheta (\delta ,\xi )=\vartheta_{0}\left(\delta ,\xi \right)+p^{1}\vartheta_{1}\left(\delta ,\xi \right)+p^{2}\vartheta_{2}\left(\delta ,\xi \right)++p^{3}\vartheta_{3}\left(\delta ,\xi \right)+\cdots .$$ (8)

Consider *p* = 1, the approximate solution of Eq (1) yields as

$$\vartheta (\delta ,\xi )=\underset{N\to \infty }{\text{lim}}\sum_{n=0}^{N}\vartheta_{n}\left(\delta ,\xi \right).$$ (9)

We may implement this idea to check its authenticity under some nonlinear fractional differential problems.

## 4 Numerical applications

This section presents some numerical applications to reveal the reliability and accuracy of MHITS. We note that this technique is more straightforward and relatively easy in providing the series solutions than previous schemes. We also look at how various surface solutions behave physically. The numerical computations and plot distributions are made by Mathematica software 11. The error distribution is displayed to show the performance of our proposed scheme. This small error clearly states that our scheme is authentic and accurate.

### 4.1 Example 1

Consider a fractional diffusion problem in two-dimensional form

$$\begin{array}{c} \frac{\partial^{\alpha }\vartheta }{\partial \xi^{\alpha }}=\frac{\partial^{2}\vartheta }{\partial \delta^{2}}+\frac{\partial^{2}\vartheta }{\partial \theta^{2}}-\vartheta , \end{array}$$ (10)

with initial

$$\begin{array}{c} \vartheta (\delta ,\theta ,0)=\text{sin}\;\delta \;\text{cos}\;\theta , \end{array}$$ (11)

and boundary conditions

$$\begin{array}{c} \begin{array}{cc} \vartheta (\delta ,0,\xi ) & =-\vartheta \left(\delta ,\pi ,\xi \right)=e^{-3\xi }\;\text{sin}\;\delta ,\\ \vartheta (0,\theta ,\xi ) & =\vartheta (\pi ,\theta ,\xi )=0. \end{array} \end{array}$$ (12)

We take the MIT

$$\begin{array}{c} \text{M}[\frac{\partial \vartheta }{\partial \xi }]=\text{M}[\frac{\partial^{2}\vartheta }{\partial \delta^{2}}+\frac{\partial^{2}\vartheta }{\partial \theta^{2}}-\vartheta ], \end{array}$$

which means

$$\begin{array}{c} \omega^{\alpha }\left[R\left(\omega \right)-\omega \vartheta \left(0\right)\right]=\text{M}[\frac{\partial^{2}\vartheta }{\partial \delta^{2}}+\frac{\partial^{2}\vartheta }{\partial \theta^{2}}-\vartheta ]. \end{array}$$

Thus, *R*(*ω*) obtained as

$$\begin{array}{c} R\left[\omega \right]=\omega \vartheta \left(0\right)+\frac{1}{\omega^{\alpha }}\text{M}[\frac{\partial^{2}\vartheta }{\partial \delta^{2}}+\frac{\partial^{2}\vartheta }{\partial \theta^{2}}-\vartheta ]. \end{array}$$

Utilizing inverse MIT, we determine

$$\begin{array}{c} \vartheta \left(\delta ,\theta ,\xi \right)=\vartheta \left(\delta ,\theta ,0\right)+{\text{M}}^{-1}[\frac{1}{\omega^{\alpha }}\text{M}[\frac{\partial^{2}\vartheta }{\partial \delta^{2}}+\frac{\partial^{2}\vartheta }{\partial \theta^{2}}-\vartheta ]. \end{array}$$

Now, apply HPS to get the He’s components

$$\begin{array}{c} \sum_{i=0}^{\infty }p^{i}\vartheta \left(\delta ,\theta ,\xi \right)=\vartheta \left(\delta ,\theta ,0\right)+{\text{M}}^{-1}[\frac{1}{\omega^{\alpha }}\text{M}[\sum_{i=0}^{\infty }p^{i}\frac{\partial^{2}\vartheta_{i}}{\partial \delta^{2}}+\sum_{i=0}^{\infty }p^{i}\frac{\partial^{2}\vartheta_{i}}{\partial \theta^{2}}-\sum_{i=0}^{\infty }p^{i}\vartheta ]. \end{array}$$ (13)

Equating *p* on both sides, we have

$$\begin{array}{cc} p^{0} & :\vartheta_{0}\left(\delta ,\theta ,\xi \right)=\vartheta \left(\delta ,\theta ,0\right)=\text{sin}\;\delta \;\text{cos}\;\theta ,\\ p^{1} & :\vartheta_{1}\left(\delta ,\theta ,\xi \right)={\text{M}}^{-1}[\frac{1}{\omega^{\alpha }}\text{M}\{\frac{\partial^{2}\vartheta_{0}}{\partial \delta^{2}}+\frac{\partial^{2}\vartheta_{0}}{\partial \theta^{2}}-\vartheta_{0}\}]=-\frac{3\xi^{\alpha }}{\Gamma (\alpha +1)}\;\text{sin}\;\delta \;\text{cos}\;\theta ,\\ p^{2} & :\vartheta_{2}\left(\delta ,\theta ,\xi \right)={\text{M}}^{-1}[\frac{1}{\omega^{\alpha }}\text{M}\{\frac{\partial^{2}\vartheta_{1}}{\partial \delta^{2}}+\frac{\partial^{2}\vartheta_{1}}{\partial \theta^{2}}-\vartheta_{1}\}]=\frac{{\left(3\xi^{\alpha }\right)}^{2}}{\Gamma (2\alpha +1)}\;\text{sin}\;\delta \;\text{cos}\;\theta ,\\ p^{3} & :\vartheta_{3}\left(\delta ,\theta ,\xi \right)={\text{M}}^{-1}[\frac{1}{\omega^{\alpha }}\text{M}\{\frac{\partial^{2}\vartheta_{2}}{\partial \delta^{2}}+\frac{\partial^{2}\vartheta_{2}}{\partial \theta^{2}}-\vartheta_{2}\}]=-\frac{{\left(3\xi^{\alpha }\right)}^{3}}{\Gamma (3\alpha +1)}\;\text{sin}\;\delta \;\text{cos}\;\theta ,\\ p^{4} & :\vartheta_{4}\left(\delta ,\theta ,\xi \right)={\text{M}}^{-1}[\frac{1}{\omega^{\alpha }}\text{M}\{\frac{\partial^{2}\vartheta_{3}}{\partial \delta^{2}}+\frac{\partial^{2}\vartheta_{3}}{\partial \theta^{2}}-\vartheta_{3}\}]=\frac{{\left(3\xi^{\alpha }\right)}^{4}}{\Gamma (4\alpha +1)}\;\text{sin}\;\delta \;\text{cos}\;\theta ,\\ & \vdots . \end{array}$$

In the same way, we can derive the following series

$$\begin{array}{c} \begin{array}{cc} \vartheta (\delta ,\theta ,\xi ) & =\vartheta_{0}\left(\delta ,\theta ,\xi \right)+\vartheta_{1}\left(\delta ,\theta ,\xi \right)+\vartheta_{2}\left(\delta ,\theta ,\xi \right)+\vartheta_{3}\left(\delta ,\theta ,\xi \right)+\vartheta_{4}\left(\delta ,\theta ,\xi \right)+\cdots ,\\ & =\text{sin}\;\delta \;\text{cos}\;\theta -\frac{3\xi^{\alpha }}{\Gamma (\alpha +1)}\;\text{sin}\;\delta \;\text{cos}\;\theta +\frac{{\left(3\xi^{\alpha }\right)}^{2}}{\Gamma (2\alpha +1)}\;\text{sin}\;\delta \;\text{cos}\;\theta \\ & -\frac{{\left(3\xi^{\alpha }\right)}^{3}}{\Gamma (3\alpha +1)}\;\text{sin}\;\delta \;\text{cos}\;\theta +\frac{{\left(3\xi^{\alpha }\right)}^{4}}{\Gamma (4\alpha +1)}\;\text{sin}\;\delta \;\text{cos}\;\theta +\cdots . \end{array} \end{array}$$ (14)

The series in Eq (14) becomes to the precise solution at *α* = 1 such as

$$\begin{array}{c} \vartheta \left(\delta ,\theta ,\xi \right)=e^{-3\xi }\;\text{sin}\;\delta \;\text{cos}\;\theta . \end{array}$$ (15)

**Fig 1:**
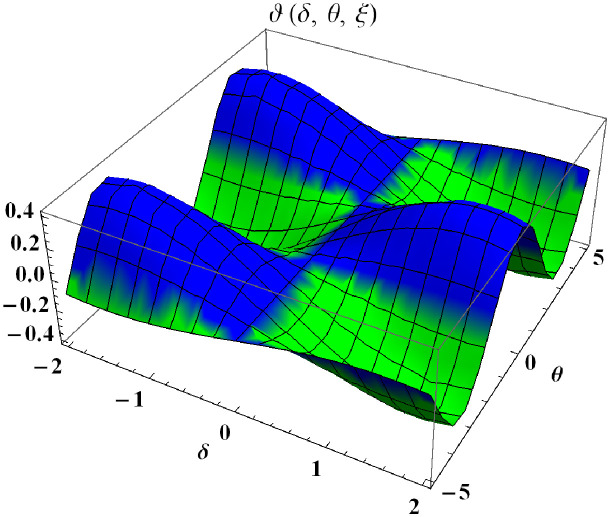
Plot distribution of numerical results.

**Fig 2:**
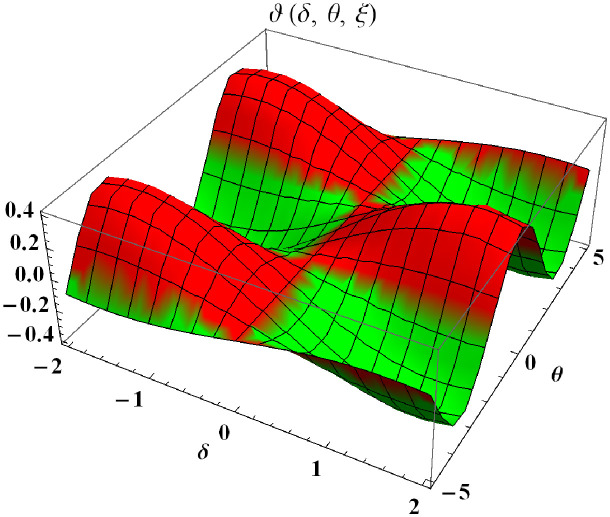
Plot distribution of precise results.

**Fig 3:**
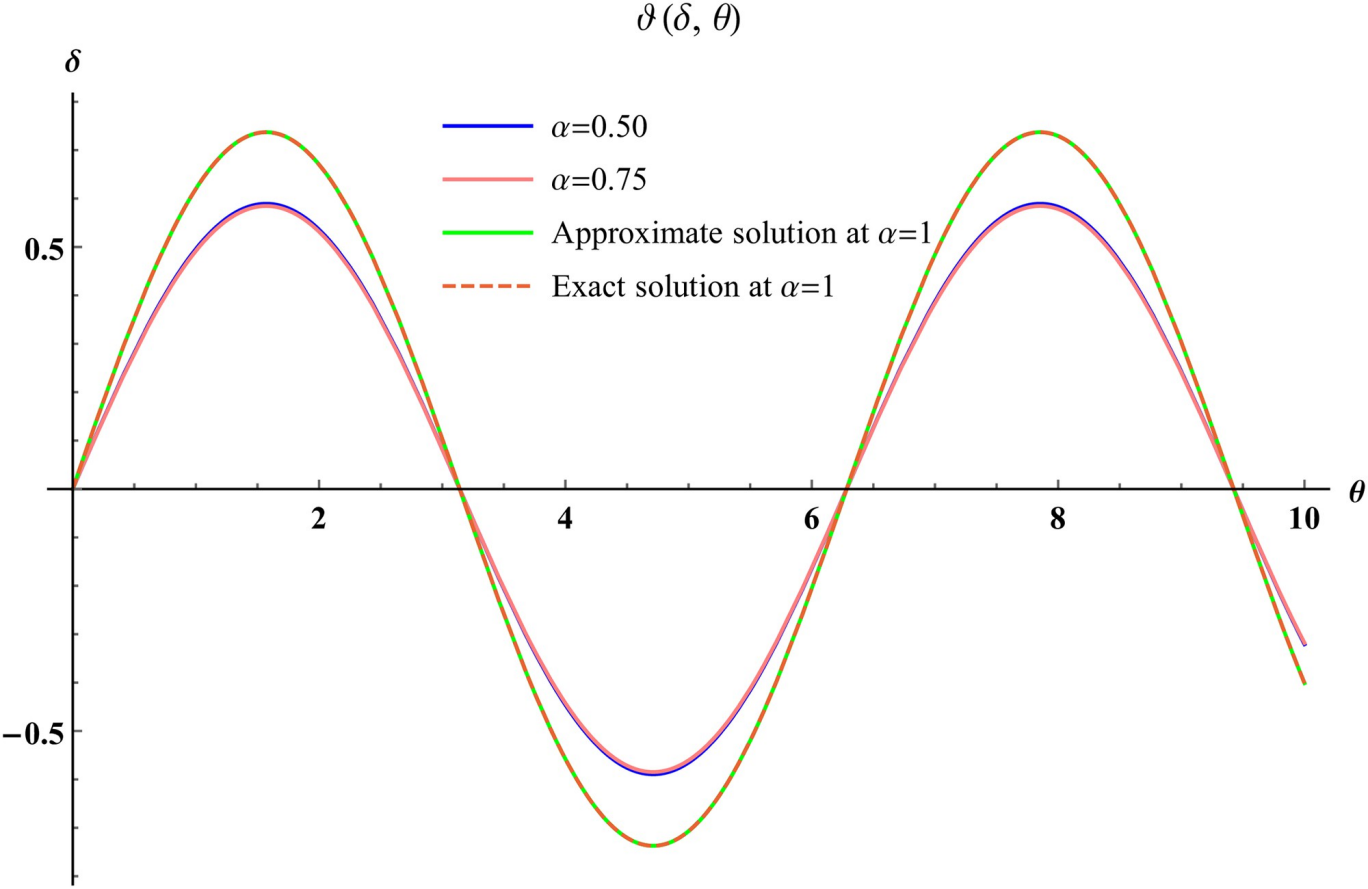
2D visual comparison between precise and MHITS results at different fractional order.

In a three dimensional case, we have plot distribution of numerical results and plot distribution of precise results. The 3D visual in Fig 1 is obtained by our proposed scheme whereas the 3D visual in Fig 2 represents the exact solution. We consider −2 ≤ *δ* ≤ 2 and −5 ≤ *θ* ≤ 5 *α* = 1. Fig 3 represents the 2D plot distribution at 0 ≤ *ϑ* ≤ 10, and shows a graphical comparison between the exact results and the MHITS solutions of fractional order *α* = 0.50, 0.75, 1. It is noted that the approximate results obtained by MHITS and the exact results have strong agreement agreement at *α* = 1.

### 4.2 Example 2

Suppose another fractional diffusion problem in two-dimensional form

$$\begin{array}{c} \frac{\partial^{\alpha }\vartheta }{\partial \xi^{\alpha }}=\frac{\partial^{2}\vartheta }{\partial \delta^{2}}+\frac{\partial^{2}\vartheta }{\partial \theta^{2}}+\text{sin}\;\theta , \end{array}$$ (16)

with initial

$$\begin{array}{c} \vartheta (\delta ,\theta ,0)=\text{sin}\;\delta \;\text{sin}\;\theta +\text{sin}\;\theta , \end{array}$$ (17)

and boundary conditions

$$\begin{array}{c} \begin{array}{cc} \vartheta (\delta ,0,\xi ) & =\vartheta (\delta ,\pi ,\xi )=0,\\ \vartheta (0,\theta ,\xi ) & =\vartheta (\pi ,\theta ,\xi )=\text{sin}\;\theta . \end{array} \end{array}$$ (18)

We take the MIT

$$\begin{array}{c} \text{M}[\frac{\partial \vartheta }{\partial \xi }]=\text{M}[\frac{\partial^{2}\vartheta }{\partial \delta^{2}}+\frac{\partial^{2}\vartheta }{\partial \theta^{2}}+\text{sin}\;\theta ], \end{array}$$

it yields

$$\begin{array}{c} \omega^{\alpha }\left[R\left(\omega \right)-\omega \vartheta \left(0\right)\right]=\text{M}[\frac{\partial^{2}\vartheta }{\partial \delta^{2}}+\frac{\partial^{2}\vartheta }{\partial \theta^{2}}+\text{sin}\;\theta ]. \end{array}$$

Thus, *R*(*ω*) obtained as

$$\begin{array}{cc} R[\omega ] & =\omega \vartheta \left(0\right)+\frac{1}{\omega^{\alpha }}\text{M}[\frac{\partial^{2}\vartheta }{\partial \delta^{2}}+\frac{\partial^{2}\vartheta }{\partial \theta^{2}}+\text{sin}\;\theta ],\\ R[\omega ] & =\omega \vartheta \left(0\right)+\frac{1}{\omega^{\alpha }}\text{M}\left[\;\text{sin}\;\theta \right]+\frac{1}{\omega^{\alpha }}\text{M}[\frac{\partial^{2}\vartheta }{\partial \delta^{2}}+\frac{\partial^{2}\vartheta }{\partial \theta^{2}}]. \end{array}$$

Utilizing inverse MIT, we determine

$$\begin{array}{c} \vartheta \left(\delta ,\theta ,\xi \right)=\vartheta \left(\delta ,\theta ,0\right)+\frac{\xi^{\alpha }}{\Gamma (\alpha +1)}\;\text{sin}\;\theta +{\text{M}}^{-1}[\frac{1}{\omega^{\alpha }}\text{M}[\frac{\partial^{2}\vartheta }{\partial \delta^{2}}+\frac{\partial^{2}\vartheta }{\partial \theta^{2}}]. \end{array}$$

Now, apply HPS to get the He’s elements

$$\begin{array}{c} \sum_{i=0}^{\infty }p^{i}\vartheta \left(\delta ,\theta ,\xi \right)=\vartheta \left(\delta ,\theta ,0\right)+\frac{\xi^{\alpha }}{\Gamma (\alpha +1)}\;\text{sin}\;\theta +{\text{M}}^{-1}[\frac{1}{\omega^{\alpha }}\text{M}[\sum_{i=0}^{\infty }p^{i}\frac{\partial^{2}\vartheta_{i}}{\partial \delta^{2}}+\sum_{i=0}^{\infty }p^{i}\frac{\partial^{2}\vartheta_{i}}{\partial \theta^{2}}]. \end{array}$$ (19)

Equating *p* on both sides, we have

$$\begin{array}{cc} p^{0}:\vartheta_{0}\left(\delta ,\theta ,\xi \right) & =\vartheta \left(\delta ,\theta ,0\right)=\text{sin}\;\delta \;\text{sin}\;\theta +\text{sin}\;\theta +\frac{\xi^{\alpha }}{\Gamma (\alpha +1)}\;\text{sin}\;\theta ,\\ p^{1}:\vartheta_{1}\left(\delta ,\theta ,\xi \right) & ={\text{M}}^{-1}[\frac{1}{\omega^{\alpha }}\text{M}\{\frac{\partial^{2}\vartheta_{0}}{\partial \delta^{2}}+\frac{\partial^{2}\vartheta_{0}}{\partial \theta^{2}}\}]\\ & =-2\frac{\xi^{\alpha }}{\Gamma (\alpha +1)}\;\text{sin}\;\delta \;\text{sin}\;\theta -\frac{\xi^{\alpha }}{\Gamma (\alpha +1)}\;\text{sin}\;\theta -\frac{{\left(\xi^{\alpha }\right)}^{2}}{\Gamma (2\alpha +1)}\;\text{sin}\;\theta ,\\ p^{2}:\vartheta_{2}\left(\delta ,\theta ,\xi \right) & ={\text{M}}^{-1}[\frac{1}{\omega^{\alpha }}\text{M}\{\frac{\partial^{2}\vartheta_{1}}{\partial \delta^{2}}+\frac{\partial^{2}\vartheta_{1}}{\partial \theta^{2}}\}]\\ & =\frac{{\left(2\xi^{\alpha }\right)}^{2}}{\Gamma (2\alpha +1)}\;\text{sin}\;\delta \;\text{sin}\;\theta +\frac{{\left(\xi^{\alpha }\right)}^{2}}{\Gamma (2\alpha +1)}\;\text{sin}\;\theta +\frac{{\left(\xi^{\alpha }\right)}^{3}}{\Gamma (3\alpha +1)}\;\text{sin}\;\theta ,\\ p^{3}:\vartheta_{3}\left(\delta ,\theta ,\xi \right) & ={\text{M}}^{-1}[2\frac{1}{\omega^{\alpha }}\text{M}\{\frac{\partial^{2}\vartheta_{2}}{\partial \delta^{2}}+\frac{\partial^{2}\vartheta_{2}}{\partial \theta^{2}}\}]\\ & =-\frac{{\left(2\xi^{\alpha }\right)}^{3}}{\Gamma (3\alpha +1)}\;\text{sin}\;\delta \;\text{sin}\;\theta -\frac{{\left(\xi^{\alpha }\right)}^{3}}{\Gamma (3\alpha +1)}\;\text{sin}\;\theta -\frac{{\left(\xi^{\alpha }\right)}^{4}}{\Gamma (4\alpha +1)}\;\text{sin}\;\theta ,\\ p^{4}:\vartheta_{4}\left(\delta ,\theta ,\xi \right) & ={\text{M}}^{-1}[\frac{1}{\omega^{\alpha }}\text{M}\{\frac{\partial^{2}\vartheta_{3}}{\partial \delta^{2}}+\frac{\partial^{2}\vartheta_{3}}{\partial \theta^{2}}\}]\\ & =\frac{{\left(2\xi^{\alpha }\right)}^{4}}{\Gamma (4\alpha +1)}\;\text{sin}\;\delta \;\text{sin}\;\theta +\frac{{\left(\xi^{\alpha }\right)}^{4}}{\Gamma (4\alpha +1)}\;\text{sin}\;\theta +\frac{{\left(\xi^{\alpha }\right)}^{5}}{\Gamma (5\alpha +1)}\;\text{sin}\;\theta ,\\ & \vdots . \end{array}$$

In the same way, we can derive the following series

$$\begin{array}{c} \begin{array}{cc} \vartheta (\delta ,\theta ,\xi ) & =\vartheta_{0}\left(\delta ,\theta ,\xi \right)+\vartheta_{1}\left(\delta ,\theta ,\xi \right)+\vartheta_{2}\left(\delta ,\theta ,\xi \right)+\vartheta_{3}\left(\delta ,\theta ,\xi \right)+\vartheta_{4}\left(\delta ,\theta ,\xi \right)+\cdots ,\\ & =\text{sin}\;\theta +\text{sin}\;\delta \;\text{sin}\;\theta (1-\frac{2\xi^{\alpha }}{\Gamma (\alpha +1)}+\frac{{\left(2\xi^{\alpha }\right)}^{2}}{\Gamma (2\alpha +1)}-\frac{{\left(2\xi^{\alpha }\right)}^{3}}{\Gamma (3\alpha +1)}+\frac{{\left(2\xi^{\alpha }\right)}^{4}}{\Gamma (4\alpha +1)}+\cdots )+\text{small}\;\text{terms}. \end{array} \end{array}$$ (20)

The series in Eq (20) becomes to the precise solution at *α* = 1 such as

$$\begin{array}{c} \vartheta \left(\delta ,\theta ,\xi \right)=\text{sin}\;\theta +e^{-2\xi }\;\text{sin}\;\delta \;\text{sin}\;\theta . \end{array}$$ (21)

**Fig 4:**
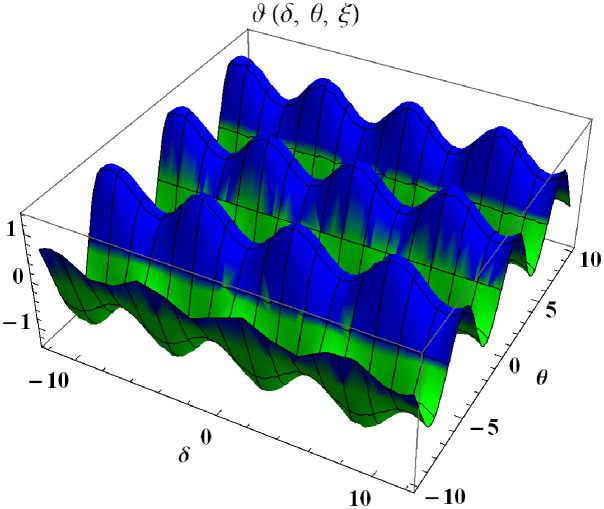
Plot distribution of numerical results.

**Fig 5:**
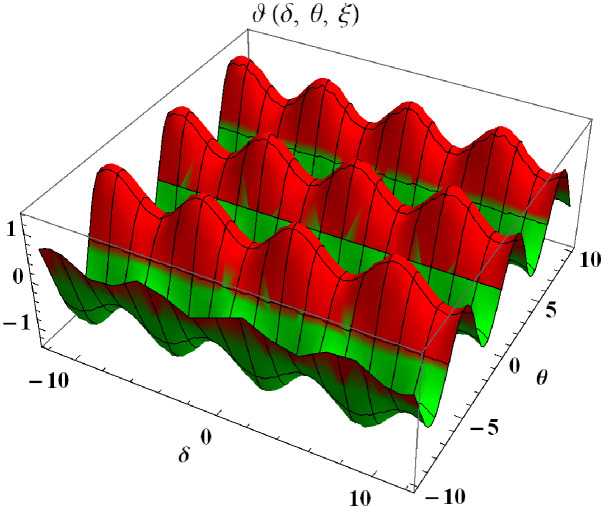
Plot distribution of precise results.

**Fig 6:**
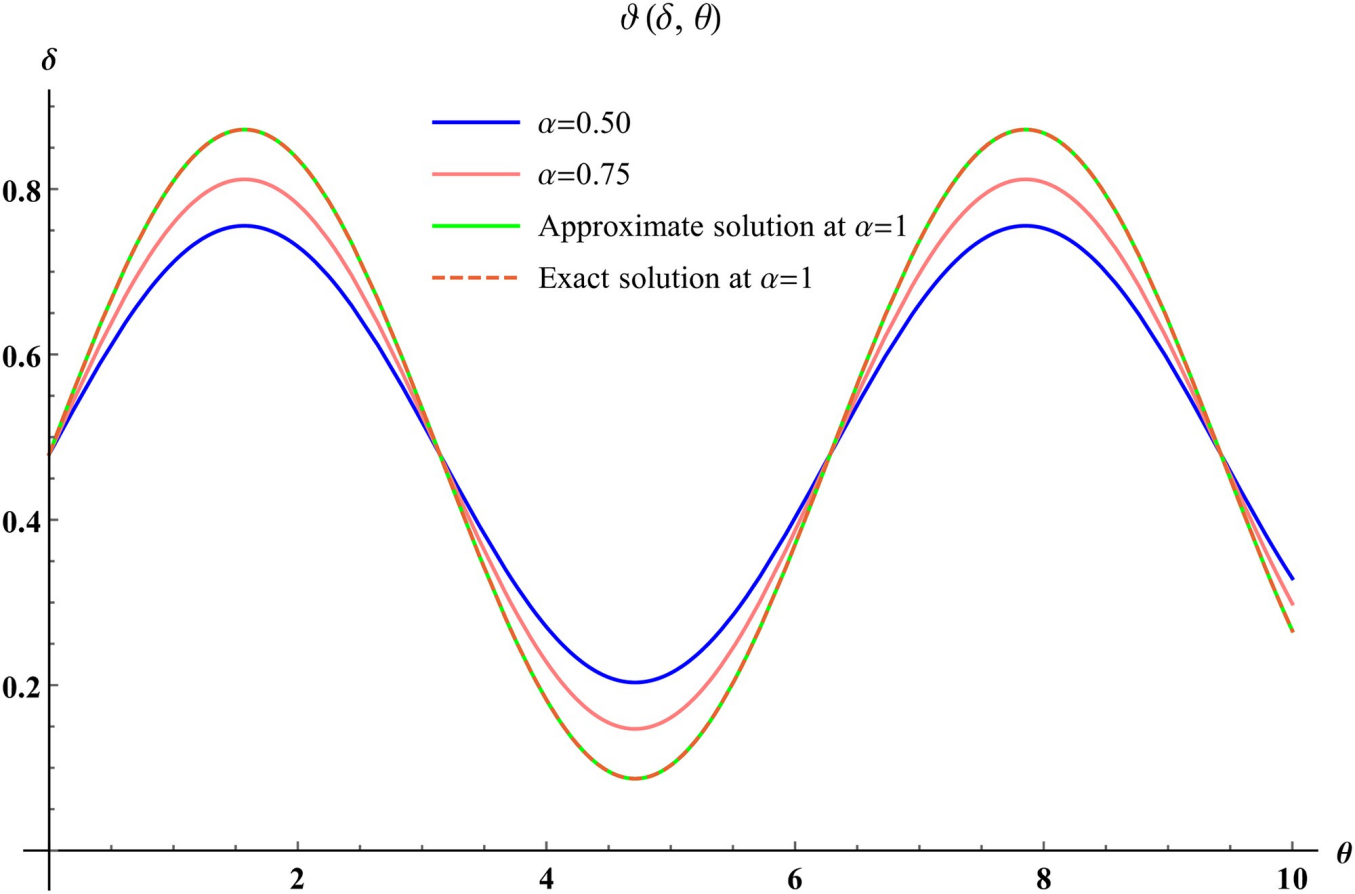
2D visual comparison between precise and MHITS results at different fractional order.

In a three dimensional case, we have plot distribution of numerical results and plot distribution of precise results. The 3D visual in Fig 4 is obtained by our proposed scheme whereas the 3D visual in Fig 5 represents the exact solution. We consider −10 ≤ *δ* ≤ 10 and −10 ≤ *θ* ≤ 10 at *α* = 1. Fig 6 represents the 2D plot distribution at 0 ≤ *ϑ* ≤ 10, and shows a graphical comparison between the exact results and the MHITS solutions of fractional order *α* = 0.50, 0.75, 1. It is noted that the approximate results obtained by MHITS and the exact results have strong agreement at *α* = 1.

### 4.3 Example 3

Consider a fractional diffusion problem in three-dimensional form

$$\begin{array}{c} \frac{\partial^{\alpha }\vartheta }{\partial \xi^{\alpha }}=\frac{\partial^{2}\vartheta }{\partial \delta^{2}}+\frac{\partial^{2}\vartheta }{\partial \theta^{2}}+\frac{\partial^{2}\vartheta }{\partial \kappa^{2}}-2\vartheta , \end{array}$$ (22)

with initial

$$\begin{array}{c} \vartheta (\delta ,\theta ,\kappa ,0)=\text{sin}\;\delta \;\text{sin}\;\theta \;\text{sin}\;\kappa , \end{array}$$ (23)

and boundary conditions

$$\begin{array}{c} \begin{array}{cc} \vartheta (0,\theta ,\kappa ,\xi ) & =\vartheta (\pi ,\theta ,\kappa ,\xi )=0,\\ \vartheta (\delta ,0,\kappa ,\xi ) & =\vartheta (\delta ,\pi ,\kappa ,\xi )=0,\\ \vartheta (\delta ,\theta ,0,\xi ) & =\vartheta (\delta ,\theta ,\pi ,\xi )=0. \end{array} \end{array}$$ (24)

Utilizing the steps of MHITS, we obtain

$$\begin{array}{c} \vartheta \left(\delta ,\theta ,\kappa ,\xi \right)=\vartheta \left(\delta ,\theta ,0\right)+{\text{M}}^{-1}[\frac{1}{\omega^{\alpha }}\text{M}[\frac{\partial^{2}\vartheta }{\partial \delta^{2}}+\frac{\partial^{2}\vartheta }{\partial \theta^{2}}+\frac{\partial^{2}\vartheta }{\partial \kappa^{2}}-2\vartheta ]. \end{array}$$

Now, apply HPS to get the He’s elements

$$\begin{array}{c} \sum_{i=0}^{\infty }p^{i}\vartheta \left(\delta ,\theta ,\kappa ,\xi \right)=\vartheta \left(\delta ,\theta ,\kappa ,0\right)+{\text{M}}^{-1}[\frac{1}{\omega^{\alpha }}\text{M}[\sum_{i=0}^{\infty }p^{i}\frac{\partial^{2}\vartheta_{i}}{\partial \delta^{2}}+\sum_{i=0}^{\infty }p^{i}\frac{\partial^{2}\vartheta_{i}}{\partial \theta^{2}}+\sum_{i=0}^{\infty }p^{i}\frac{\partial^{2}\vartheta_{i}}{\partial \kappa^{2}}-2\sum_{i=0}^{\infty }p^{i}\vartheta ]. \end{array}$$ (25)

Equating *p* on both sides, we have

$$\begin{array}{cc} p^{0} & :\vartheta_{0}\left(\delta ,\theta ,\kappa ,\xi \right)=\vartheta \left(\delta ,\theta ,\kappa ,0\right)=\text{sin}\;\delta \;\text{sin}\;\theta \;\text{sin}\;\kappa ,\\ p^{1} & :\vartheta_{1}\left(\delta ,\theta ,\xi \right)={\text{M}}^{-1}[\frac{1}{\omega^{\alpha }}\text{M}\{\frac{\partial^{2}\vartheta_{0}}{\partial \delta^{2}}+\frac{\partial^{2}\vartheta_{0}}{\partial \theta^{2}}+\frac{\partial^{2}\vartheta_{3}}{\partial \kappa^{2}}-2\vartheta_{0}\}]=-\frac{5\xi^{\alpha }}{\Gamma (\alpha +1)}\;\text{sin}\;\delta \;\text{sin}\;\theta \;\text{sin}\;\kappa ,\\ p^{2} & :\vartheta_{2}\left(\delta ,\theta ,\xi \right)={\text{M}}^{-1}[\frac{1}{\omega^{\alpha }}\text{M}\{\frac{\partial^{2}\vartheta_{1}}{\partial \delta^{2}}+\frac{\partial^{2}\vartheta_{1}}{\partial \theta^{2}}+\frac{\partial^{2}\vartheta_{3}}{\partial \kappa^{2}}-2\vartheta_{1}\}]=\frac{{\left(5\xi^{\alpha }\right)}^{2}}{\Gamma (2\alpha +1)}\;\text{sin}\;\delta \;\text{sin}\;\theta \;\text{sin}\;\kappa ,\\ p^{3} & :\vartheta_{3}\left(\delta ,\theta ,\xi \right)={\text{M}}^{-1}[2\frac{1}{\omega^{\alpha }}\text{M}\{\frac{\partial^{2}\vartheta_{2}}{\partial \delta^{2}}+\frac{\partial^{2}\vartheta_{2}}{\partial \theta^{2}}+\frac{\partial^{2}\vartheta_{3}}{\partial \kappa^{2}}-2\vartheta_{2}\}]=-\frac{{\left(5\xi^{\alpha }\right)}^{3}}{\Gamma (3\alpha +1)}\;\text{sin}\;\delta \;\text{sin}\;\theta \;\text{sin}\;\kappa ,\\ p^{4} & :\vartheta_{4}\left(\delta ,\theta ,\xi \right)={\text{M}}^{-1}[\frac{1}{\omega^{\alpha }}\text{M}\{\frac{\partial^{2}\vartheta_{3}}{\partial \delta^{2}}+\frac{\partial^{2}\vartheta_{3}}{\partial \theta^{2}}+\frac{\partial^{2}\vartheta_{3}}{\partial \kappa^{2}}-2\vartheta_{3}\}]=\frac{{\left(5\xi^{\alpha }\right)}^{4}}{\Gamma (4\alpha +1)}\;\text{sin}\;\delta \;\text{sin}\;\theta \;\text{sin}\;\kappa ,\\ & \vdots . \end{array}$$

likewise, we may deduce the subsequent sequence as follows:

$$\begin{array}{c} \begin{array}{cc} \vartheta (\delta ,\theta ,\kappa ,\xi ) & =\vartheta_{0}\left(\delta ,\theta ,\xi \right)+\vartheta_{1}\left(\delta ,\theta ,\xi \right)+\vartheta_{2}\left(\delta ,\theta ,\xi \right)+\vartheta_{3}\left(\delta ,\theta ,\xi \right)+\vartheta_{4}\left(\delta ,\theta ,\xi \right)+\cdots ,\\ & =\text{sin}\;\delta \;\text{sin}\;\theta \;\text{sin}\;\kappa -\frac{5\xi^{\alpha }}{\Gamma (\alpha +1)}\;\text{sin}\;\delta \;\text{sin}\;\theta \;\text{sin}\;\kappa +\frac{{\left(5\xi^{\alpha }\right)}^{2}}{\Gamma (2\alpha +1)}\;\text{sin}\;\delta \;\text{sin}\;\theta \;\text{sin}\;\kappa \\ & -\frac{{\left(5\xi^{\alpha }\right)}^{3}}{\Gamma (3\alpha +1)}\;\text{sin}\;\delta \;\text{sin}\;\theta \;\text{sin}\;\kappa +\frac{{\left(5\xi^{\alpha }\right)}^{4}}{\Gamma (4\alpha +1)}\;\text{sin}\;\delta \;\text{sin}\;\theta \;\text{sin}\;\kappa +\cdots . \end{array} \end{array}$$ (26)

The series in Eq (26) becomes to the precise solution at *α* = 1 such as

$$\begin{array}{c} \vartheta \left(\delta ,\theta ,\kappa ,\xi \right)=e^{-5\xi }\;\text{sin}\;\delta \;\text{sin}\;\theta \;\text{sin}\;\kappa . \end{array}$$ (27)

**Fig 7:**
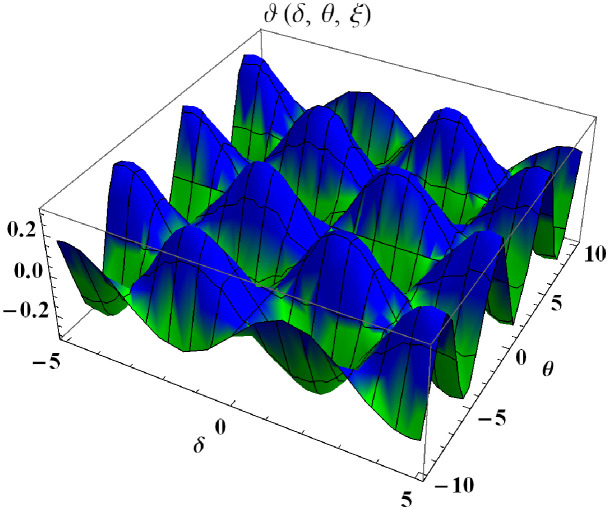
Plot distribution of numerical results.

**Fig 8:**
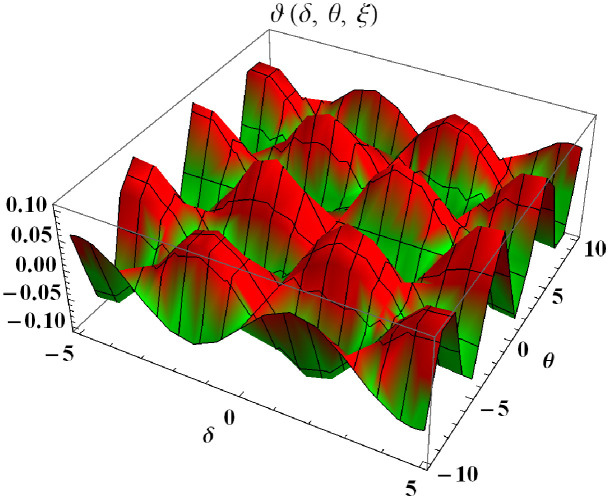
Plot distribution of precise results.

**Fig 9:**
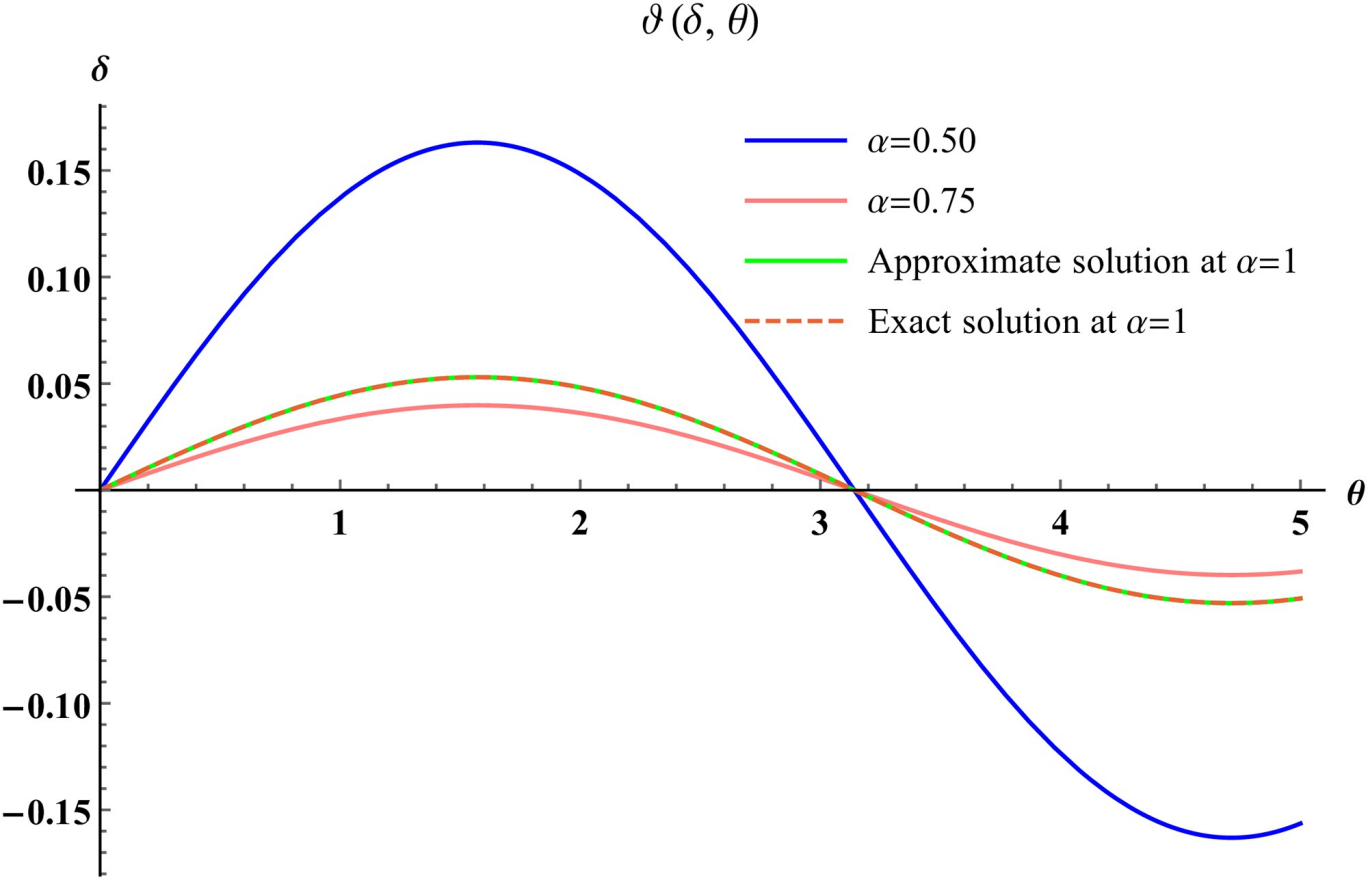
2D visual comparison between precise and MHITS results at different fractional order.

In a three dimensional case, we have plot distribution of numerical results and plot distribution of precise results. The 3D visual in Fig 7 is obtained by our proposed scheme whereas the 3D visual in Fig 8 represents the exact solution. We consider −5 ≤ *δ* ≤ 5 and −10 ≤ *θ* ≤ 10 at *α* = 1. Fig 9 represents the 2D plot distribution at 0 ≤ *ϑ* ≤ 5, and shows a graphical comparison between the exact results and the MHITS solutions of fractional order *α* = 0.50, 0.75, 1. It is noted that the approximate results obtained by MHITS and the exact results have strong agreement at *α* = 1.

### 4.4 Example 4

Consider a fractional diffusion problem in three-dimensional form

$$\begin{array}{c} \frac{\partial^{\alpha }\vartheta }{\partial \xi^{\alpha }}=\frac{\partial^{2}\vartheta }{\partial \delta^{2}}+\frac{\partial^{2}\vartheta }{\partial \theta^{2}}+\frac{\partial^{2}\vartheta }{\partial \kappa^{2}}+\text{sin}\;\kappa , \end{array}$$ (28)

with initial

$$\begin{array}{c} \vartheta (\delta ,\theta ,\kappa ,0)=\text{sin}(\delta +\theta )+\text{sin}\;\kappa , \end{array}$$ (29)

and boundary conditions

$$\begin{array}{c} \begin{array}{cc} \vartheta (0,\theta ,\kappa ,\xi ) & =\vartheta \left(\pi ,\theta ,\kappa ,\xi \right)=\text{sin}\;\kappa +e^{-2\xi }\;\text{sin}\;\theta ,\\ \vartheta (\delta ,0,\kappa ,\xi ) & =\vartheta \left(\delta ,\pi ,\kappa ,\xi \right)=\text{sin}\;\kappa +e^{-2\xi }\;\text{sin}\;\delta ,\\ \vartheta (\delta ,\theta ,0,\xi ) & =\vartheta \left(\delta ,\theta ,\pi ,\xi \right)=e^{-2\xi }\;\text{sin}\left(\delta +\theta \right). \end{array} \end{array}$$ (30)

Utilizing the steps of MHITS, we obtain

$$\begin{array}{c} \vartheta \left(\delta ,\theta ,\kappa ,\xi \right)=\vartheta \left(\delta ,\theta ,\kappa ,0\right)+\frac{\xi^{\alpha }}{\Gamma (\alpha +1)}\;\text{sin}\;\kappa +{\text{M}}^{-1}[\frac{1}{\omega^{\alpha }}\text{M}[\frac{\partial^{2}\vartheta }{\partial \delta^{2}}+\frac{\partial^{2}\vartheta }{\partial \theta^{2}}+\frac{\partial^{2}\vartheta }{\partial \kappa^{2}}]. \end{array}$$

Now, apply HPS to get the He’s elements

$$\begin{array}{c} \sum_{i=0}^{\infty }p^{i}\vartheta \left(\delta ,\theta ,\kappa ,\xi \right)=\vartheta \left(\delta ,\theta ,\kappa ,0\right)+\frac{\xi^{\alpha }}{\Gamma (\alpha +1)}\;\text{sin}\;\kappa +{\text{M}}^{-1}[\frac{1}{\omega^{\alpha }}\text{M}[\sum_{i=0}^{\infty }p^{i}\frac{\partial^{2}\vartheta_{i}}{\partial \delta^{2}}+\sum_{i=0}^{\infty }p^{i}\frac{\partial^{2}\vartheta_{i}}{\partial \theta^{2}}+\sum_{i=0}^{\infty }p^{i}\frac{\partial^{2}\vartheta_{i}}{\partial \kappa^{2}}]. \end{array}$$ (31)

Equating *p* on both sides, we have

$$\begin{array}{cc} p^{0}:\vartheta_{0}\left(\delta ,\theta ,\kappa ,\xi \right) & =\vartheta \left(\delta ,\theta ,0\right)=\text{sin}\left(\delta +\theta \right)+\text{sin}\;\kappa +\frac{\xi^{\alpha }}{\Gamma (\alpha +1)}\;\text{sin}\;\kappa ,\\ p^{1}:\vartheta_{1}\left(\delta ,\theta ,\kappa ,\xi \right) & ={\text{M}}^{-1}[\frac{1}{\omega^{\alpha }}\text{M}\{\frac{\partial^{2}\vartheta_{0}}{\partial \delta^{2}}+\frac{\partial^{2}\vartheta_{0}}{\partial \theta^{2}}\}]\\ & =-\frac{2\xi^{\alpha }}{\Gamma (\alpha +1)}\text{sin}\left(\delta +\theta \right)-\xi^{\alpha }\;\text{sin}\;\kappa -\frac{{\left(\xi^{\alpha }\right)}^{2}}{\Gamma (2\alpha +1)}\;\text{sin}\;\kappa ,\\ p^{2}:\vartheta_{2}\left(\delta ,\theta ,\kappa ,\xi \right) & ={\text{M}}^{-1}[\frac{1}{\omega^{\alpha }}\text{M}\{\frac{\partial^{2}\vartheta_{1}}{\partial \delta^{2}}+\frac{\partial^{2}\vartheta_{1}}{\partial \theta^{2}}\}]\\ & =\frac{{\left(2\xi^{\alpha }\right)}^{2}}{\Gamma (2\alpha +1)}\text{sin}\left(\delta +\theta \right)+\frac{{\left(\xi^{\alpha }\right)}^{2}}{\Gamma (2\alpha +1)}\;\text{sin}\;\kappa +\frac{{\left(\xi^{\alpha }\right)}^{3}}{\Gamma (3\alpha +1)}\;\text{sin}\;\kappa ,\\ p^{3}:\vartheta_{3}\left(\delta ,\theta ,\kappa ,\xi \right) & ={\text{M}}^{-1}[2\frac{1}{\omega^{\alpha }}\text{M}\{\frac{\partial^{2}\vartheta_{2}}{\partial \delta^{2}}+\frac{\partial^{2}\vartheta_{2}}{\partial \theta^{2}}\}]\\ & =-\frac{{\left(2\xi^{\alpha }\right)}^{3}}{\Gamma (3\alpha +1)}\text{sin}\left(\delta +\theta \right)-\frac{{\left(\xi^{\alpha }\right)}^{3}}{\Gamma (3\alpha +1)}\;\text{sin}\;\kappa -\frac{{\left(\xi^{\alpha }\right)}^{4}}{\Gamma (4\alpha +1)}\;\text{sin}\;\kappa ,\\ p^{4}:\vartheta_{4}\left(\delta ,\theta ,\kappa ,\xi \right) & ={\text{M}}^{-1}[\frac{1}{\omega^{\alpha }}\text{M}\{\frac{\partial^{2}\vartheta_{3}}{\partial \delta^{2}}+\frac{\partial^{2}\vartheta_{3}}{\partial \theta^{2}}\}]\\ & =\frac{{\left(2\xi^{\alpha }\right)}^{4}}{\Gamma (4\alpha +1)}\text{sin}\left(\delta +\theta \right)+\frac{{\left(\xi^{\alpha }\right)}^{4}}{\Gamma (4\alpha +1)}\;\text{sin}\;\kappa +\frac{{\left(\xi^{\alpha }\right)}^{5}}{\Gamma (5\alpha +1)}\;\text{sin}\;\kappa ,\\ & \vdots . \end{array}$$

likewise, we may deduce the subsequent sequence as follows:

$$\begin{array}{c} \begin{array}{cc} \vartheta (\delta ,\theta ,\kappa ,\xi ) & =\vartheta_{0}\left(\delta ,\theta ,\kappa ,\xi \right)+\vartheta_{1}\left(\delta ,\theta ,\kappa ,\xi \right)+\vartheta_{2}\left(\delta ,\theta ,\kappa ,\xi \right)+\vartheta_{3}\left(\delta ,\theta ,\kappa ,\xi \right)+\vartheta_{4}\left(\delta ,\theta ,\kappa ,\xi \right)+\cdots ,\\ & =\text{sin}\;\kappa +\text{sin}\left(\delta +\theta \right)(1-\frac{2\xi^{\alpha }}{\Gamma (\alpha +1)}+\frac{{\left(2\xi^{\alpha }\right)}^{2}}{\Gamma (2\alpha +1)}-\frac{{\left(2\xi^{\alpha }\right)}^{3}}{\Gamma (3\alpha +1)}+\frac{{\left(2\xi^{\alpha }\right)}^{4}}{\Gamma (4\alpha +1)}-\frac{{\left(2\xi^{\alpha }\right)}^{5}}{\Gamma (5\alpha +1)}+\cdots )+\text{noise}\;\text{terms}. \end{array} \end{array}$$ (32)

The series in Eq (32) becomes to the precise solution at *α* = 1 such as

$$\begin{array}{c} \vartheta \left(\delta ,\theta ,\kappa ,\xi \right)=\text{sin}\;\kappa +e^{-2\xi }\text{sin}\left(\delta +\theta \right). \end{array}$$ (33)

**Fig 10:**
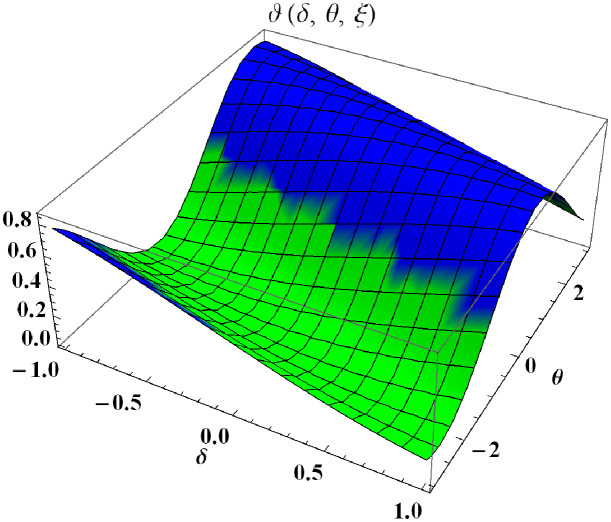
Plot distribution of numerical results.

**Fig 11:**
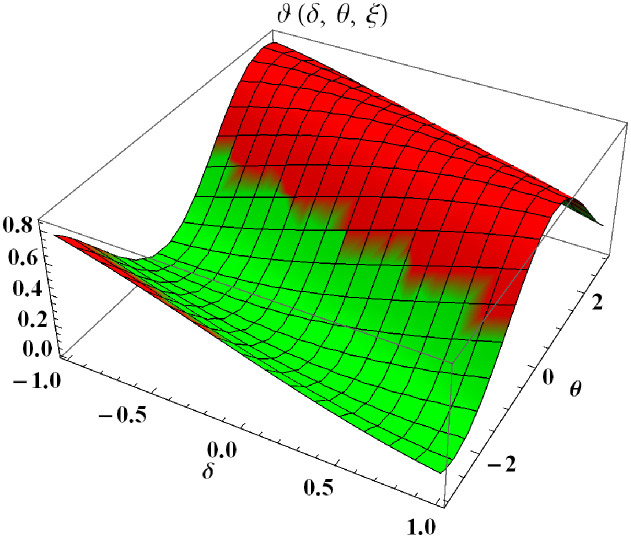
Plot distribution of precise results.

**Fig 12:**
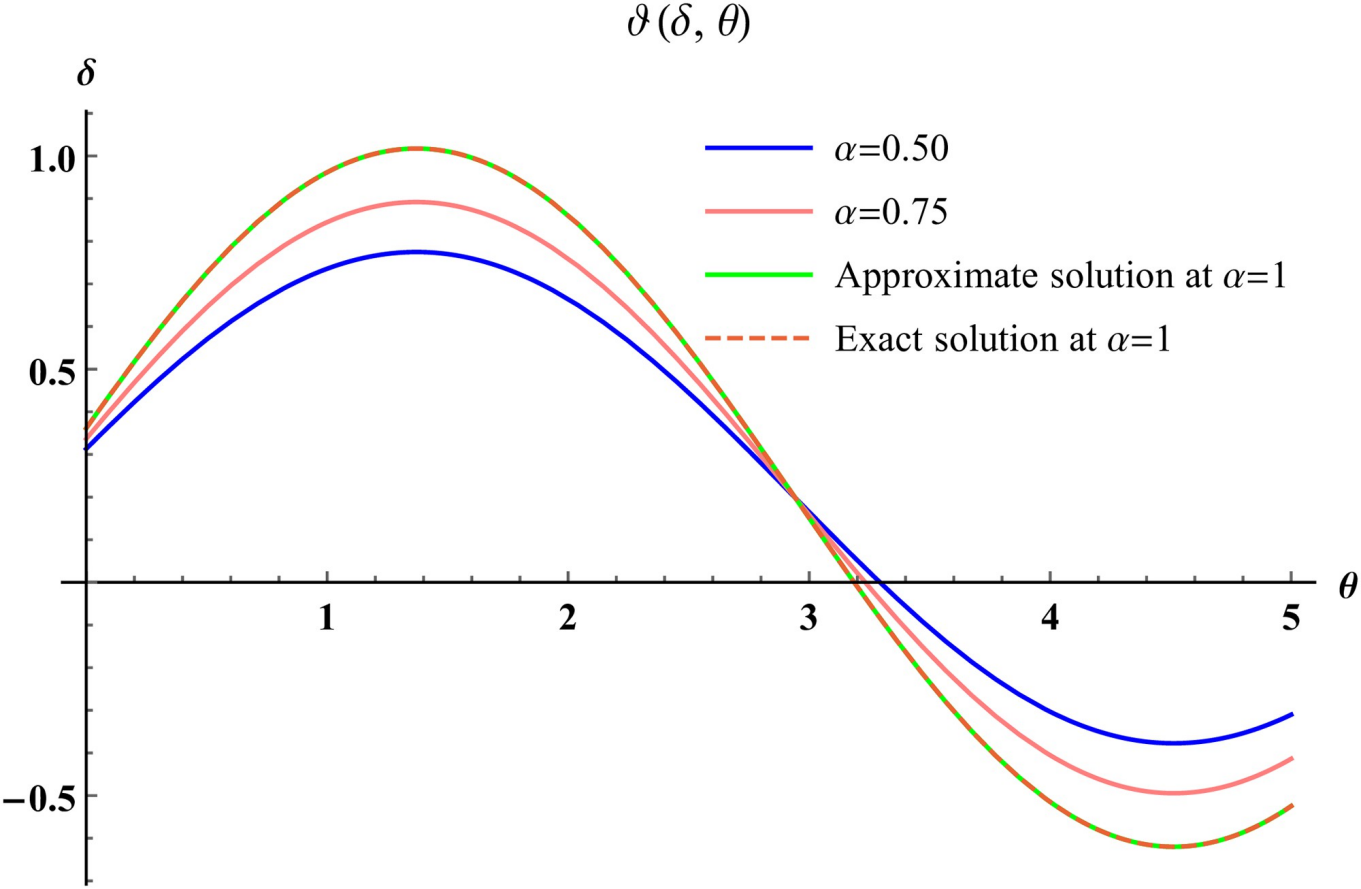
2D visual comparison between precise and MHITS results at different fractional order.

In a three dimensional case, we have plot distribution of numerical results and plot distribution of precise results. The 3D visual in Fig 10 is obtained by our proposed scheme whereas the 3D visual in Fig 11 represents the exact solution. We consider −1 ≤ *δ* ≤ 1 and −3 ≤ *θ* ≤ 10 at *α* = 1. Fig 12 represents the 2D plot distribution at 0 ≤ *ϑ* ≤ 5, and shows a graphical comparison between the exact results and the MHITS solutions of fractional order *α* = 0.50, 0.75, 1. It is noted that the approximate results obtained by MHITS and the exact results have strong agreement at *α* = 1.

## 5 Conclusion

In this study, we have systematically developed an innovative strategy of MHITS and utilized it to achieve the numerical results of two-dimensional and three-dimensional fractional problems that arise in heat flows. By employing this method, the computational effort required to locate the solution in power series form can be minimized, where the coefficients are determined in a consecutive algebraic step. This strategy is capable of handling the recurrence relation which is free from the implementation of integration and restrictions of variables that may face some assumptions. The MIT has the advantage of direct implementation whereas HPS produced the results in the form of series. This series yields the approximate results and converges to the precise results very quickly. The iterators can be readily computed using the concept of the limit as the variable approaches infinity. This approach is also applicable to a broad range of FPDEs in physics and engineering. In the future, we plan to apply this scheme to other fractional problems such as fractional Cauchy reaction-diffusion problems, time-fractional Ginzburg-Landau model, time-fractional Noyes-Field model, and other fractional problems of science and technology.
